# Metagenomics and Metagenome-Assembled Genomes Analysis of Highland Barley Baijiu Daqu

**DOI:** 10.3390/microorganisms14040877

**Published:** 2026-04-14

**Authors:** Lihua Chen, Yuhang Chen, Qinghua Peng, Dingxia Zhou, Shengbao Feng

**Affiliations:** 1 Faculty of Flavour Fragrance and Cosmetics, Shanghai Institute of Technology, Shanghai 201418, China; 13739185299@163.com (Y.C.); pengqinghua0322@163.com (Q.P.); 18930465101@163.com (D.Z.); 2 Qinghai Huzhu Barley Wine Co., Ltd., Haidong 810500, China

**Keywords:** highland barley, Baijiu, Daqu, metagenomics, community structure, metagenome-assembled genomes

## Abstract

Highland barley Baijiu is a kind of fermented liquor with national characteristics produced in the Qinghai–Tibet Plateau, and its quality largely depends on the highland barley Baijiu Daqu (HBQ). HBQ contains abundant microbial resources and embedded unknown genomes that have not yet been decoded. In order to deeply understand the key contribution of microorganisms in HBQ, this study analyzed the microbial community structure of HBQ, inferred predicted functions and recovered high-quality metagenome-assembled genomes (MAGs) based on Metagenomics. The results indicated that *Pantoea agglomerans* was the most abundant species in HBQ, followed by *Lichtheimia ramosa*, *Pichia kudriavzevii*, *Saccharomycopsis fibuligera* and *Wickerhamomyces anomalus*. The predictive function of the HBQ was focused on annotating carbohydrate metabolism and amino acid metabolism. Meanwhile, six high-quality MAG strains were recovered and identified as *Unclassified Kroppenstedtia*, *Erwinia persicina*, *Leuconostoc citreum*, *Saccharopolyspora rectivirgula*, *Levilactobacillus brevis*, and *Pantoea agglomerans*. Genome annotation of the recovered genomes showed eggNOG predicted function as well as primary and secondary metabolites. The metabolic network diagram of the functional microorganisms in HBQ related to flavor compounds was also predicted. The results can help to understand the formation mechanism of flavor profiles in highland barley Baijiu.

## 1. Introduction

Highland barley Baijiu is a traditional distilled alcoholic beverage, which is believed to originate from the Qinghai–Tibet Plateau [1]. The unique climatic conditions of this region, including mild winters, cool summers, and abundant sunshine, provide inherent advantages for the production of Highland Baijiu. It is closely associated with local food culture and is commonly consumed on important social occasions such as festivals, weddings and banquets. As Highland barley, the principal fermentation substrate of Highland barley Baijiu, is rich in protein, dietary fiber, vitamins, and β-glucan [2], Highland barley-based fermented products have nutritional and regional significance.

Like other types of Chinese Baijiu, highland barley Baijiu is produced through multi-round solid-state fermentation [3]. The process generally involves grain steaming, Daqu-assisted saccharification and fermentation, distillation, and post-processing steps. In this process, Highland barley Baijiu Daqu (HBQ) serves as the key starter and is regarded as a major determinant of Baijiu quality [4]. HBQ introduces the essential enzymes and microorganisms to drive the fermentation, thus producing ethyl acetate, ethyl lactate, phenethyl alcohol and other aroma substances responsible for the characteristic flavor of highland barley Baijiu [5]. Therefore, understanding the microbiota of Daqu is essential for elucidating the fermentation behavior and flavor quality of Highland barley Baijiu.

The application of metagenomics in microbial sequencing of Daqu has been relatively mature, which can provide more accurate microbial species annotation and functional insights [6]. The microorganisms involved in Daqu fermentation mainly include bacteria, yeasts, and filamentous fungi, and these groups play complementary roles in substrate degradation and flavor formation [7]. Bacterial members commonly reported in Baijiu Daqu include lactic acid bacteria such as *Lactobacillus*, *Leuconostoc*, and *Weissella*, as well as *Bacillus* and other heat-tolerant taxa; fungal members include molds such as *Aspergillus*, *Rhizopus*, *Rhizomucor*, *Lichtheimia*, and *Thermoascus*, together with yeasts such as *Saccharomyces*, *Pichia*, *Saccharomycopsis*, and *Wickerhamomyces*. In general, molds contribute hydrolytic enzymes for starch and protein degradation, yeasts participate in ethanol production and ester precursor formation, whereas bacteria are important for acid production, amino acid transformation, and other flavor-related metabolic processes.

MAG-based analysis is a genome-resolved extension of metagenomic analysis, which can uncover previously unexplored microbial resources through assembly and binning [8]. A MAG is a complete or near-complete microbial genome reconstructed directly from environmental DNA by assembling short reads into contigs and then grouping contigs from the same microbial population through binning. This strategy is particularly suitable for complex microbial ecosystems such as HBQ, where some microorganisms adapted to the plateau environment may be difficult to isolate by conventional cultivation [9]. Therefore, MAG-level analysis enables culture-independent investigation of the metabolic potential of individual microbial populations during Highland barley Baijiu fermentation.

Despite the importance of HBQ in the brewing of Highland barley Baijiu, its microbial community structure and genome-resolved functional potential remain insufficiently understood, and studies involving metagenomics and MAG recovery in HBQ are still limited. We hypothesized that mature HBQ harbors a relatively stable core microbiota with distinct functional potential related to carbohydrate metabolism, and that genome-resolved metagenomic analysis would provide genome-level insight into functionally relevant microbial populations within this fermentation ecosystem. In this study, shotgun metagenomic sequencing was applied to mature HBQ to characterize its microbial community structure, annotate its predicted functions using multiple databases, recover high-quality MAGs, and infer the potential contributions of functional microorganisms to flavor formation in Highland barley Baijiu.

## 2. Materials and Methods

### 2.1. Daqu Production Process

**Figure 1:**
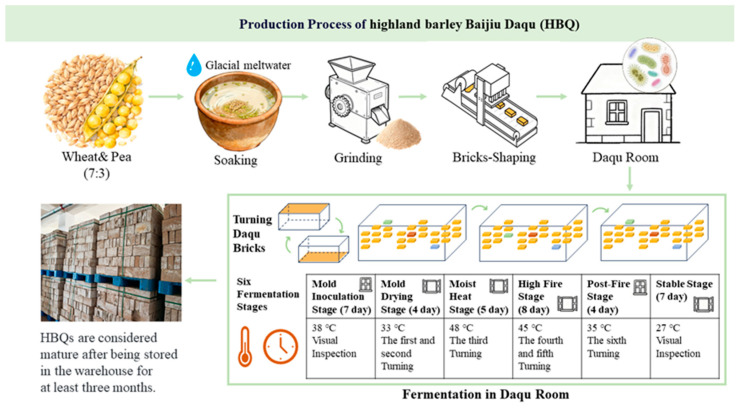
The production process of highland barley Baijiu Daqu. Soaking, grinding, and brick shaping are the pretreatment steps in HBQ production. The shaped bricks are then placed in the Daqu room, where fermentation and microbial enrichment occur. The Daqu-room process consists of six fermentation stages, and the corresponding process parameters are shown in the relevant parts of the figure.

The production process of HBQ is shown in Figure 1. At first, mature wheat and peas were soaked thoroughly in glacier meltwater, crushed, and pressed into brick shapes. Then, the HBQ bricks were placed in the Daqu room for about 35 days. During this period, the maturation process of the HBQ was gradually promoted through six stages, including adjusting temperature, ventilation, and flipping HBQ bricks according to different requirements. After fermentation in the Daqu room, HBQ became mature in the warehouse for three to six months.

### 2.2. Sample Collection

HBQ (Batch No. 20230920) was supplied by Qinghai Huzhu Barley Wine Co., Ltd. The Daqu was prepared from wheat and pea (Figure 1). HBQ samples were collected at the mature stage (the Daqu bricks had been stored in the warehouse for three months) and had passed routine quality inspection by professional workers. According to the sampling strategy shown in Figure S1, samples were obtained from multiple mature HBQ bricks collected from different positions in the Daqu room. A total of six parallel subsamples were prepared for metagenomic sequencing to improve sample representativeness and assess reproducibility. After collection, the sampled bricks were crushed, homogenized, and stored at −80 °C prior to DNA extraction.

### 2.3. Metagenome DNA Extraction and Shotgun Sequencing

For each metagenomic library, 0.5 g of Daqu powder was used for total microbial genomic DNA extraction. DNA was extracted using the MagBeads FastDNA Kit for Soil (116564384) (MP Biomedicals, Irvine, CA, USA) according to the manufacturer’s instructions and stored at −20 °C prior to further assessment. The quantity and quality of extracted DNAs were measured using a QubitTM 4 Fluorometer, with WiFi: Q33238 (Invitrogen, Carlsbad, CA, USA) and agarose gel electrophoresis, respectively. The extracted microbial DNA was processed to construct metagenome shotgun sequencing libraries with insert sizes of 400 bp by using the Illumina TruSeq Nano DNA LT Library Preparation Kit. Each library was sequenced by Illumina NovaSeq X Plus (Illumina, San Diego, CA, USA) with a PE150 strategy.

### 2.4. Bioinformatics Analysis

The sequence data processing was conducted as described according to [10]. Sequence reads were first quality-filtered using fastp (Version 0.23.2), in which low-quality bases (Q < 20) were trimmed and reads shorter than 50 bp were discarded. Then reads aligned to the pea, wheat and human genomes (GCA_024323335.2; GCA_904849725.1; GCA_000001405.29) were removed from the raw data set by minimap2 (version 2.24-r1122). The remaining high-quality reads of each DNA sample were assembled by MEGAHIT (version 1.1.2). Species annotation was conducted on the acquired non-redundant protein set using MMseqs2 (version 13.45111), with a database comprising prokaryotic and eukaryotic microbial sequences from NCBI-nr and viral sequences from RVDB. Gene functional annotation was performed using MMseqs2 (parameter -s 5.7) and Diamond (version 2.0.15, blastp mode, --sensitive; e-value ≤ 0.001, sequence identity ≥ 60%) against multiple databases, including Kyoto Encyclopedia of Genes and Genomes (KEGG, http://www.kegg.jp/kegg/, accessed on 7 November 2025), Evolutionary genealogy of genes, Non-supervised Orthologous Groups (eggNOG, http://eggnogdb.embl.de/, accessed on 13 November 2025), Carbohydrate Active enzymes Database (CAZy, http://www.cazy.org/, accessed on 16 November 2025), Gene Ontology (GO, http://geneontology.org/, accessed on 20 November 2025), and MetaCyc databases (https://metacyc.org/, accessed on 29 November 2025).

### 2.5. Construction of Metagenome-Assembled Genomes (MAGs)

The subsampling of sequencing reads was performed using the fast, lightweight FASTA manipulation program seqtk (https://github.com/lh3/seqtk, accessed on 2 December 2025) if applicable. Subsequently, binning was carried out on the assembled contigs to construct metagenome-assembled genomes (MAGs) using Metabinner (version 1.4.4) [11], which applies k-means clustering on tetranucleotide frequency and coverage depth features separately and in combination, followed by a two-stage SCG-based refinement to integrate multiple clustering results. The quality of each MAG was evaluated using CheckM (version 1.1.3). Based on the resulting completeness and contamination values, genomes with >50% completeness and <10% contamination were classified as medium-quality, while those with >90% completeness and <5% contamination were designated as high-quality. Medium-to-high quality MAGs (completeness > 50%, contamination < 10%) were dereplicated using dRep (version 3.5.0) with a secondary clustering ANI threshold of 99.9% (species level, corresponding to dDDH ≥ 70%) to remove redundant species-level genomes. The resulting non-redundant MAGs were used for subsequent analysis, and taxonomic assignment was performed using GTDB-Tk (version 2.6.1).

### 2.6. Functional Prediction of the High-Quality MAG

Functional annotation of the high-quality MAG was conducted using eggNOG-mapper v2 against the eggNOG database v5.0.2. The circular view of each MAG was carried out using Proksee (https://proksee.ca/, accessed on 5 December 2025). Clustered regularly interspaced short palindromic repeats elements were also checked using the CRISPR-associated (Cas) proteins finder. Bayesian Analysis of Gene Expression Level (BAGEL) 4 (http://bagel4.molgenrug.nl/, accessed on 7 December 2025) was used to screen for secondary metabolites, and the antiSMASH bacterial version (https://antismash.secondarymetabolites.org/, accessed on 9 December 2025) was also used to screen other biosynthetic gene clusters [12].

### 2.7. Statistical Analysis

Six parallel subsamples were prepared for sequencing to assess sampling reproducibility. Alpha diversity indices analysis was performed in R (version 4.3.3) using the vegan (version 2.6.6.1). TBtools (version 1.082) software was utilized to create heatmaps. Species and functional composition bar charts, bubble plots, and contribution Sankey diagrams were created using Origin 2026 and R software (version 4.5.3). Community taxonomic composition plots were generated using Krona software (https://github.com/marbl/Krona/wiki, accessed on 25 November 2025). The metabolic network diagram was assembled using Photoshop 2019 and PowerPoint 2021.

## 3. Results

### 3.1. Statistics of Sequencing

After the total DNA of the HBQ was found to be qualified through electrophoresis detection, metagenomic sequencing was performed using the Illumina NovaSeq platform. Mean sequencing depth was 11.0 Gb (Table S1). Assembly produced, on average, 215 k contigs per sample. The minimum length contig in any given assembly was 300 bp, while the maximum length reached 253 kb, yielding total assembly lengths of 284.9 ± 29.5 mb. Clustering analysis of the sequencing sequences was carried out at a similarity level of 97%.

### 3.2. Microbial Community and Alpha Diversity Analysis

**Figure 2:**
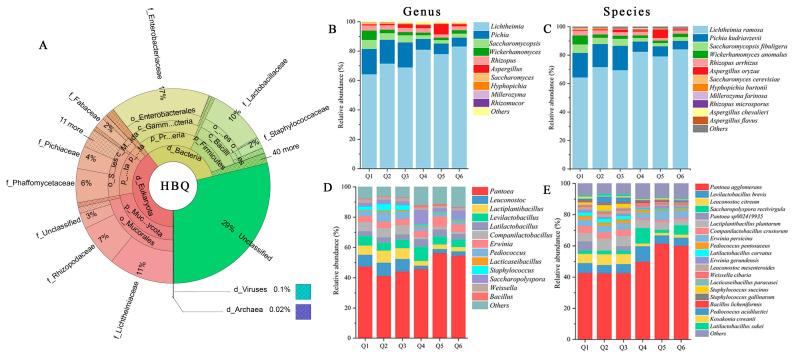
Visualization of the structural composition of colonies in HBQ at the level of kingdom, phylum, order, and family through Krona interaction diagrams (**A**). Relative abundance of fungi at the genus (**B**) and species (**C**) levels of microbial colonies in HBQ. Relative abundance of bacteria at the genus (**D**) and species (**E**) levels of microbial colonies in HBQ.

Shotgun metagenomic sequence analysis revealed the different structural domains in HBQ. Eukaryota were the most abundant microorganisms in HBQ, accounting for 39.42% ± 3.34% of the total abundance (Figure 2A). Followed by Bacteria, accounting for only 31.79% ± 3.43%. Viruses and archaea were also detected in the HBQ samples, accounting for 0.17% ± 0.09% and 0.03% ± 0.01%. The remaining 28.59% ± 0.55% of sequences were unclassified at the domain level. After removing unannotated taxa, the annotated microbial community of HBQ consisted of approximately 55% fungi and 45% bacteria. Fungi had a relative advantage in the HBQ microbial environment. Taxonomic classification revealed the presence of different domains, with a total of 87 phyla, 179 classes, 381 orders, 655 families, 1365 genes, and 2807 species identified. The sequencing depth was calculated for the alpha diversity index after rarefying to 95% of the minimum sample sequencing depth. Alpha diversity indices were calculated for each metagenomic library to describe HBQ-sample community diversity (Table S2).

#### 3.2.1. Fungal Community

After confirming that the sequencing depth was sufficient to cover the microbial diversity of HBQ, unannotated sequences were removed, and the bacterial and fungal community structures were subsequently characterized based on taxonomic annotation at the kingdom level. The fungal community of HBQ was mainly composed of *Mucoromycota* and *Ascomycota*, which together accounted for 99.04% of the total fungal relative abundance. Among them, *Mucoromycota* accounted for more than 70.80%. At the class and order levels, *Mucoromycetes* and *Mucorales* were the most abundant taxa, followed by *Saccharomycetes* and *Saccharomycetales*, respectively. At the genus level, *Lichtheimia* and *Pichia* were the two most abundant fungal genera in HBQ, with relative abundances of 67.41% and 16.50%, respectively. *Saccharomycopsis*, *Wickerhamomyces*, and *Rhizopus* showed relative abundances of 5.34%, 4.25%, and 3.28%, respectively. Analysis at the species level showed that the top 10 fungal OTUs accounted for 98.76% of the total fungal abundance in HBQ. Among them, *Lichtheimia ramosa* was the most abundant species, representing 63.49% of the total fungal abundance. *Pichia kudriavzevii*, *Saccharomycopsis fibuligera*, *Wickerhamomyces anomalus*, and *Rhizopus arrhizus* accounted for 16.94%, 5.94%, 6.19%, and 3.37%, respectively. Together, these five fungal species represented 95.93% of the total fungal abundance. In addition, *Aspergillus oryzae*, *Saccharomyces cerevisiae*, *Hyphopichia burtonii*, and *Millerozyma farinosa* were also detected in HBQ, although each accounted for less than 1% of the total fungal abundance.

#### 3.2.2. Bacterial Community

The bacterial community of HBQ was primarily composed of *Proteobacteria*, *Firmicutes*, and *Actinobacteria*, with a combined relative abundance of more than 99.95%. At lower taxonomic levels, *Enterobacteriaceae* and *Lactobacillaceae* were the dominant families, corresponding mainly to the orders *Enterobacterales* and *Lactobacillales*, and the classes *Gammaproteobacteria* and *Bacilli*, respectively. At the species level, the top 10 bacterial OTUs accounted for 86.94% of the total bacterial abundance in HBQ. Among them, *Pantoea agglomerans* showed the highest abundance, accounting for 41.34% of the total bacterial community, followed by *Levilactobacillus brevis* at 6.08%. In addition, *Leuconostoc citreum*, *Pantoea sp002419935*, *Lactiplantibacillus plantarum*, *Companilactobacillus crustorum*, and *Erwinia persicina* accounted for 5.68%, 5.58%, 5.24%, 4.32%, and 3.89%, respectively. Together, these seven bacterial taxa represented 72.13% of the total bacterial abundance in HBQ. Other detected taxa included *Pediococcus acidilactici*, *Staphylococcus succinus*, *Latilactobacillus curvatus*, *Saccharopolyspora rectivirgula*, *Pediococcus pentosaceus*, *Leuconostoc mesenteroides*, and *Weissella cibaria*, all of which were present at relatively low abundance.

### 3.3. Comparison of Microbial Community Between HBQ and Some Other Flavor-Type Baijiu Daqu

To further evaluate the taxonomic position of HBQ among Baijiu starters, the microbial composition of HBQ was compared with published microbial profiles of other flavor-type Baijiu Daqu, such as sauce-flavor Baijiu [13], strong-flavor Baijiu [14], light-flavor Baijiu [15], feng-flavor Baijiu [16], rice-flavor Baijiu [17], chi-flavor Baijiu [18] and Laobaigan-flavor Baijiu [19] (Figure S2). The clustering analysis showed that HBQ and light-flavor Baijiu Daqu were grouped together at the lowest linkage height, indicating the highest similarity in community structure between these two starter types. This cluster was subsequently linked with sauce-flavor Baijiu Daqu, whereas the other Daqu types showed greater separation from HBQ in the dendrogram.

At the genus level, HBQ showed relatively high standardized abundances of *Lichtheimia*, *Pantoea*, *Leuconostoc*, *Levilactobacillus*, *Companilactobacillus*, *Wickerhamomyces*, *Erwinia*, and *Kluyveromyces*. In comparison, sauce-flavor Daqu was characterized by relatively higher abundances of *Kroppenstedtia*, *Penicillium*, *Fusarium*, *Paenibacillus*, *Thermoactinomyces*, and *Virgibacillus*; light-flavor Daqu showed enrichment of *Isatchenkia*, *Torulaspora*, *Hyphopichia*, *Enterobacter*, and *Raoultella*; strong-flavor Daqu showed relatively higher abundances of *Acinetobacter*, *Rasamsonia*, and *Clavispora*; feng-flavor Daqu was enriched in *Saccharopolyspora*, *Bacillus*, *Streptomyces*, *Pichia*, *Thermoascus*, and *Citrobacter*; chi-flavor Daqu showed higher abundances of *Lactobacillus*, *Rhizopus*, *Thermomucor*, *Mucor*, *Kosakonia*, and *Saccharomycopsis*; rice-flavor Daqu was characterized by *Acetobacter*, *Weissella*, *Pediococcus*, *Lactococcus*, and *Saccharomyces*; and Laobaigan-flavor Daqu showed relatively higher abundances of *Isatchenkia* and *Enterobacter*.

### 3.4. Functional Annotation Based on Multiple Databases

#### 3.4.1. KEGG Database

**Figure 3:**
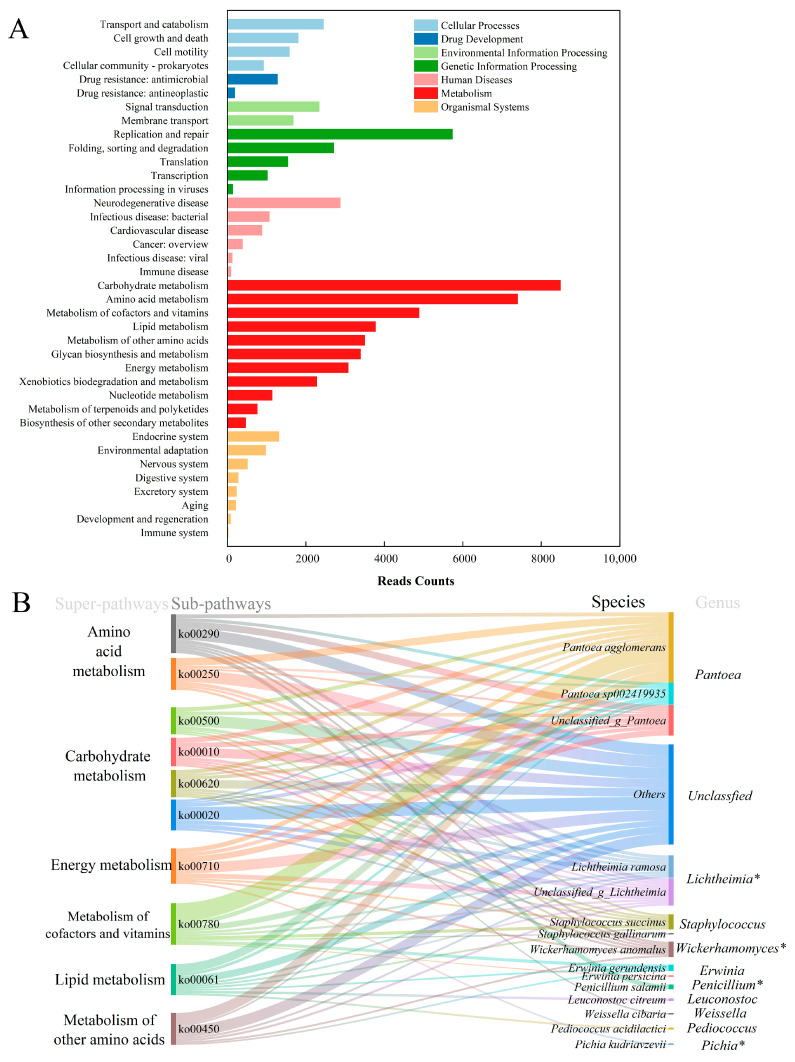
The functional annotation of microbial community genes in HBQ by KEGG (**A**). The contribution of microorganisms to metabolism (**B**): only the 10 most abundant taxonomic groups at the genus level were shown; The asterisk symbol (*) is used to denote fungal taxa.

In order to achieve a more comprehensive predictive function for the HBQ, several databases were annotated in this research. About 31.73% of HBQ metagenomic open reading frames were annotated in the KEGG database and assigned to 196 KEGG pathways, which were subsequently divided into 3 levels. Multiple biological pathways were identified (level 1), with metabolism being the predominant pathway, accounting for 54.28% (Figure 3A).

Further analysis revealed the predicted super-pathways (level 2) of genes, with carbohydrate metabolism being identified as the most dominant pathway, followed by amino acid metabolism, replication and repair, metabolism of cofactors and vitamins, and lipid metabolism, accounting for 11.89%, 10.36%, 8.03%, 6.84%, and 5.28%, respectively.

As shown in Figure 3B, the top ten pathways in terms of sub-pathways (level 3) abundance for metabolic functions were valine, leucine, and isoleucine biosynthesis (ko00290), alanine, aspartate, and glutamate metabolism (ko00250), starch and sucrose metabolism (ko00500), carbon fixation in photosynthetic organisms (ko00710), glycolysis/glycogenesis (ko00010), biotin metabolism (ko00780), Pyruvate metabolism (ko00620), citric acid cycle (TCA cycle) (ko00020), fatty acid biosynthesis (ko00061), and selenium metabolism (ko00450). To evaluate species-specific functional contributions in HBQ, a species–function association analysis was performed based on the correspondence between microbial taxa, sub-pathway metabolic functions, and their relative abundances in the samples. The results showed that genes involved in metabolic activities were mainly contributed by *Pantoea*, *Lichtheimia*, *Staphylococcus*, *Wickerhamomyces*, *Erwinia*, *Penicillium*, *Leuconostoc*, *Weissella*, *Pediococcus* and *Pichia*. Analysis of the top 10 metabolic pathways further showed that approximately 20% of the associated functions could not be assigned to identified taxa.

#### 3.4.2. eggNOG Database

**Figure 4:**
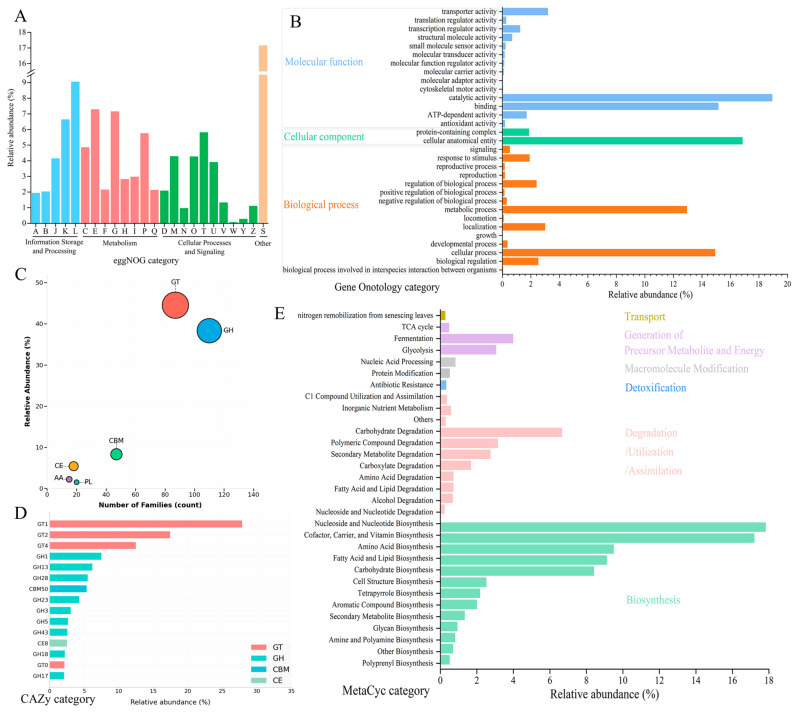
Functional annotation of HBQ microbiota genes based on eggNOG (**A**): [A] RNA processing and modification, [B] Chromatin structure and dynamics, [C] Energy production and conversion, [D] Cell cycle control, cell division, chromosome partitioning, [E] Amino acid transport and metabolism, [F] Nucleotide transport and metabolism, [G] Carbohydrate transport and metabolism, [H] Coenzyme transport and metabolism, [I] Lipid transport and metabolism, [J] Translation, ribosomal structure and biogenesis, [K] Transcription, [L] Replication, recombination and repair, [M] Cell wall/membrane/envelope biogenesis, [N] Cell motility, [O] Posttranslational modification, protein turnover, chaperones, [P] Inorganic ion transport and metabolism, [Q] Secondary metabolites biosynthesis, transport and catabolism, [S] Function unknown, [T] Signal transduction mechanisms, [U] Intracellular trafficking, secretion, and vesicular transport, [V] Defense mechanisms, [W] Extracellular structures, [Y] Nuclear structure, [Z] Cytoskeleton), MetaCyc (**B**), CAZy (**C**): family number and relative abundance; (**D**): top 15 key CAZy families), and GO (**E**).

Functional annotation based on the eggNOG database showed that the high-abundance genes in HBQ were mainly associated with carbohydrate metabolism, amino acid metabolism, and replication and repair (Figure 4A). Among these categories, carbohydrate metabolism and amino acid metabolism were the most prominent functional groups.

#### 3.4.3. Gene Ontology Database

Gene Ontology (GO) functional annotation of HBQ was shown in Figure 4B. Catalytic activity (19.2%) and binding (15.3%) were the dominant molecular functions. Cellular anatomical entity (16.8%) represented the major cellular component. Metabolic process (12.7%) and cellular process (14.5%) were the most abundant biological processes, followed by biological regulation (2.8%) and localization (3.2%).

#### 3.4.4. CAZy Database

Figure 4C shows the relationship between family number and relative abundance of six CAZy classes. GT (Glycosyltransferases, 87 families, 44.5%) and GH (Glycoside Hydrolases, 110 families, 38.3%) were the dominant classes. CBM (Carbohydrate-Binding Modules, 47 families, 8.3%), CE (Carbohydrate Esterases, 18 families, 5.4%), AA (Auxiliary Activities, 15 families, 2.2%), and PL (Polysaccharide Lyases, 20 families, 1.5%) showed lower abundances. Figure 4D presents the relative abundance of the top 15 key CAZy families. GT1 (28%), GT2 (17.5%), and GT4 (12.5%) ranked the top three. Nine GH families (including GH1, GH13, and GH28) were detected, indicating strong glycoside hydrolysis capacity. CBM50 (5.4%) and CE8 (2.5%) were also among the top 15 families.

#### 3.4.5. MetaCyc Database

The enrichment of the MetaCyc pathway in HBQ was analyzed based on the MetaCyc database (Figure 4E). Functional annotation of the HBQ sample based on MetaCyc categories revealed a dominant representation of biosynthetic processes, with the highest relative abundances observed for nucleoside and nucleotide biosynthesis (17.8%) and cofactor, carrier, and vitamin biosynthesis (17.2%), followed by amino acid biosynthesis (9.5%), fatty acid and lipid biosynthesis (9.1%), and carbohydrate biosynthesis (8.4%). Degradation, utilization, and assimilation-related pathways constituted the second most abundant functional class, where carbohydrate degradation (6.8%) was the most prominent subcategory, alongside polymeric compound degradation (3.1%), secondary metabolite degradation (2.8%), and other degradation processes. Pathways involved in the generation of precursor metabolites and energy, including fermentation (4.0%), glycolysis (3.0%), and the TCA cycle (1.0%), were detected at moderate levels. Additional functional categories accounted for less than 1.5% of the relative abundance in the HBQ.

### 3.5. Metabolic Network Predictions Related to Volatile Flavor Compounds

**Figure 5:**
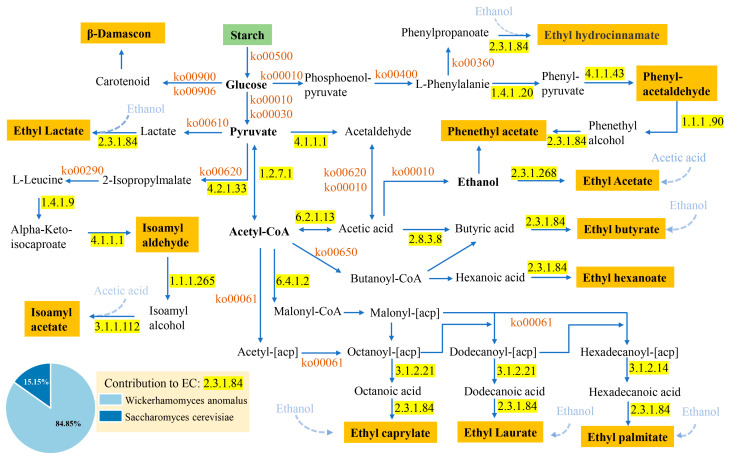
Predicted metabolic network of substrate decomposition and differential flavor compounds formation by referring to the KEGG database detected in the HBQ metagenome. The green and orange rectangles refer to substrates and differential flavor compounds, respectively.

To further clarify the specific metabolic pathways of 13 key volatile flavor compounds with OAV > 1 in highland barley Baijiu and determine their responsible microorganisms, the KEGG database and previous literature were used to predict their metabolic network (Figure 5) [3]. The predicted network included pathways related to starch degradation, glycolysis, phenylalanine metabolism, fatty acid elongation, pyruvate metabolism, and the tricarboxylic acid (TCA) cycle. Glucose, pyruvate, and acetyl-CoA were identified as central intermediates in these pathways.

**Figure 6:**
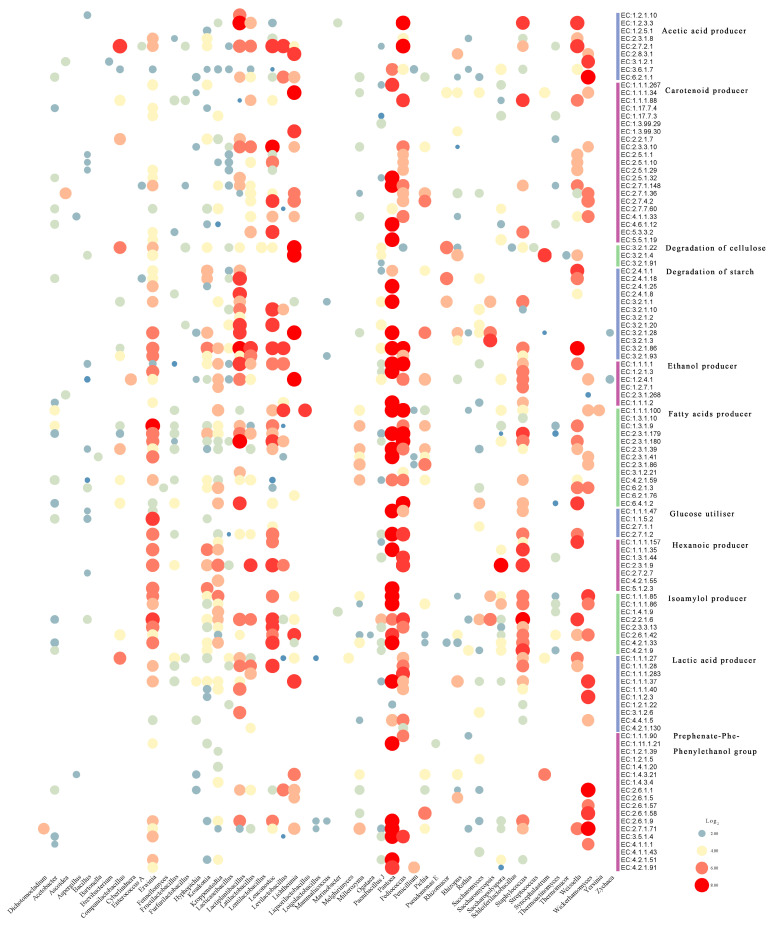
Functional microorganisms related to flavor substances and their enzyme profiles in HBQ. The diameter of the bubble correlates with the relative abundance of enzymes. Vertical coordinates indicate the EC numbers of the functional enzymes.

An abundance bubble chart was further generated to illustrate the relationships between annotated enzymes and microbial genera (Figure 6). Several enzymes were associated with only one genus. For example, *Kroppenstedtia* was the only genus associated with phenylalanine dehydrogenase (EC 1.4.1.20), *Saccharomyces* was uniquely associated with phenylpyruvate decarboxylase (EC 4.1.1.43), and *Pediococcus* was specifically associated with aryl-alcohol dehydrogenase (EC 1.1.1.90).

The predicted network suggested that phenethyl acetate and ethyl 3-phenylpropionate were mainly associated with the phenylalanine metabolism pathway (ko00360). In the leucine-related pathway, aconitate hydratase (EC 4.2.1.3) was associated with the conversion of pyruvate to 2-isopropylmalate, followed by the generation of α-ketoisocaproate through the leucine synthesis pathway. Pyruvate decarboxylase (EC 4.1.1.1), contributed by *Wickerhamomyces*, *Pichia*, *Aspergillus*, *Saccharomyces*, and *Millerozyma*, was associated with the formation of isoamyl aldehyde. However, 3-methylbutanal reductase (EC 1.1.1.265) and isoamyl acetate esterase (EC 3.1.1.112), which would be required for further conversion of isoamyl aldehyde into isoamyl acetate, were not enriched in HBQ.

The predicted formation of lipid-related volatile compounds was mainly associated with the fatty acid elongation pathway (ko00061). *Pantoea agglomerans* was the major contributor to acyl-[acyl-carrier-protein] hydrolase (EC 3.1.2.14), which was linked to palmitic acid synthesis. In addition, alcohol O-acetyltransferase (EC 2.3.1.84) was associated with *Wickerhamomyces anomalus* and *Saccharomyces cerevisiae*.

For organic acid metabolism, malate dehydrogenase (EC 1.1.1.37), associated with *Pantoea*, *Lichtheimia*, *Wickerhamomyces*, and *Pediococcus*, was linked to the conversion of malate to oxaloacetate. Malate dehydrogenase (oxaloacetate-decarboxylating) (NADP+) (EC 1.1.1.40) was associated with *Lactiplantibacillus*, *Pediococcus*, and *Staphylococcus*, and was linked to the conversion of malate and oxaloacetate to pyruvate. In addition, *Wickerhamomyces*, *Brevibacterium*, and *Ascoidea* showed a relatively high abundance of acetyl-CoA hydrolase (EC 3.1.2.1), which was associated with the hydrolysis of acetyl-CoA to acetate in pyruvate metabolism (ko00620). Lactic acid formation from pyruvate through lactate dehydrogenase (EC 1.1.1.27 and EC 1.1.1.28) was mainly associated with five lactic acid bacteria, namely *Leuconostoc citreum*, *Levilactobacillus brevis*, *Lactiplantibacillus plantarum*, *Pediococcus pentosaceus*, and *Pediococcus acidilactici*.

### 3.6. Metagenome-Assembled Genomes (MAGs)

Through gene prediction of the assembled sequences and eggNOG annotation, protein functions and taxonomic information of the microbial community were further characterized. A total of 42 medium-quality metagenome-assembled genomes (MAGs) were recovered from the HBQ metagenomic data, including 9 high-quality MAGs (Table S3). Based on mapping to the Genome Taxonomy Database, these MAGs were assigned to 18 distinct species-level clusters, among which three clusters remained unclassified. The genome size of the recovered MAGs ranged from 739 kb to 5344 kb, and the GC content ranged from 32.5% to 72.2%.

The high-quality MAGs were identified as *Unclassified Kroppenstedtia* (MAG1, MAG12, and MAG22), *Erwinia persicina* (MAG2), *Levilactobacillus brevis* (MAG4 and MAG34), *Pantoea agglomerans* (MAG14), *Saccharopolyspora rectivirgula* (MAG20), and *Leuconostoc citreum* (MAG33). *Pantoea agglomerans*, *Levilactobacillus brevis*, *Leuconostoc citreum*, *Acetobacter malorum*, and *Unclassified Kroppenstedtia* were shared among multiple binners. In addition, CRISPR/Cas proteins were detected in most MAGs (Table S4), whereas no CRISPR/Cas-related genes were detected in MAG29 and MAG37.

### 3.7. Predictive Functional Analysis of High-Quality MAGs

**Figure 7:**
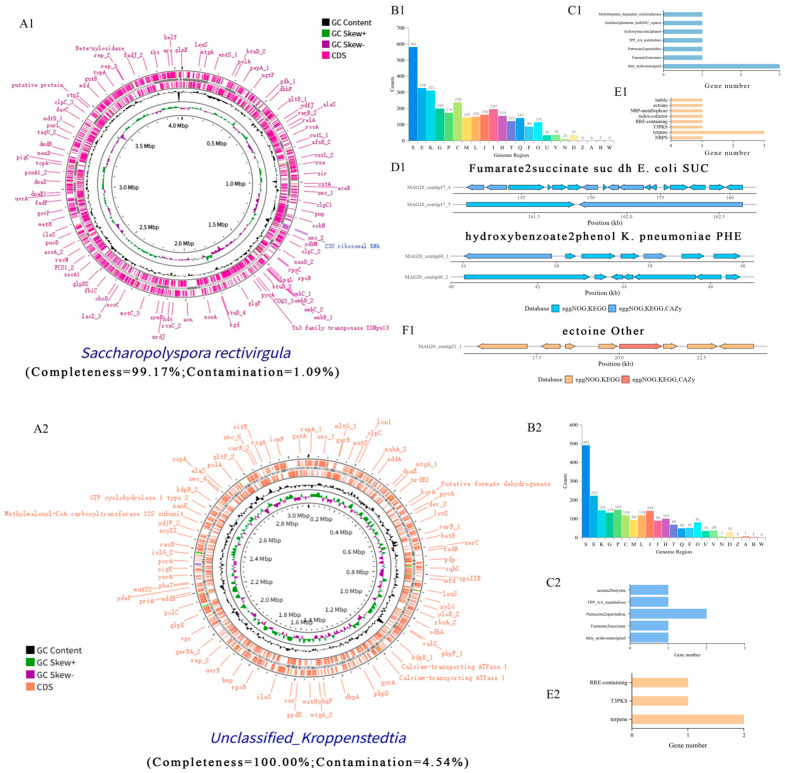

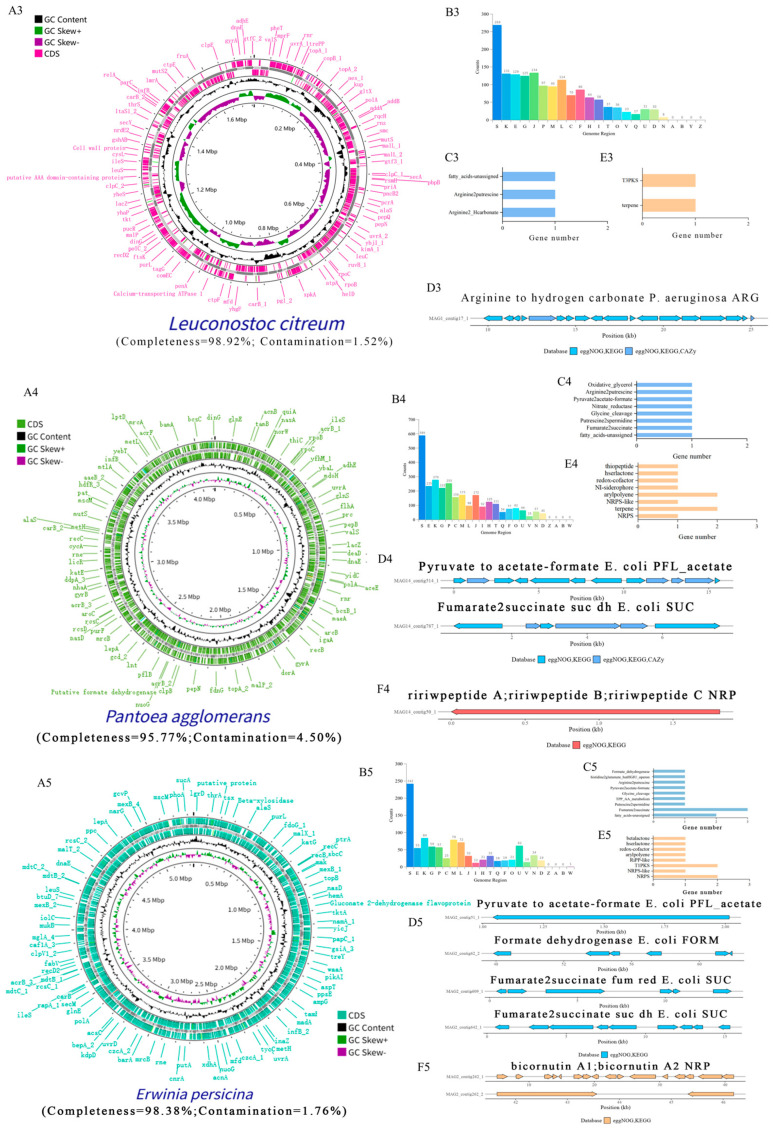

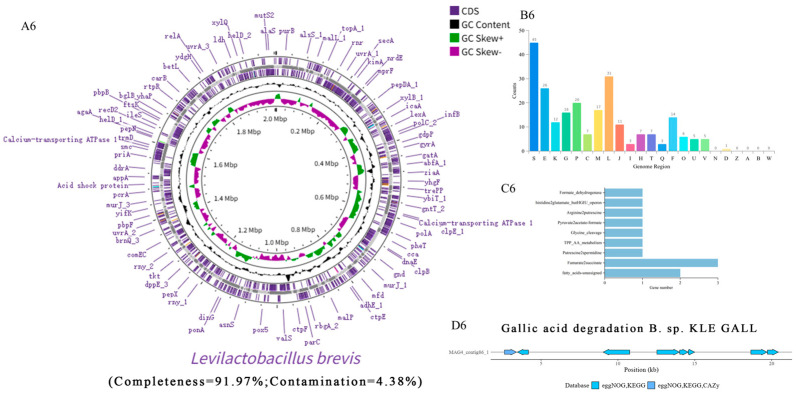
Circular genome visualization of high-quality MAGs (**A**); The functional annotation of MAGs microbiota genes by eggNOG (**B**: All letter designations are defined as in Figure 4A); The Gene number annotation of primary metabolites (**C**) and secondary metabolites (**E**); Genomic mapping enables visualization of gene distribution across contigs within each MAG, revealing regions that exhibit 100% similarity to known gene clusters (**D**: primary metabolites; **F**: secondary metabolites). Panel labels with the same number represent the same MAG.

The eggNOG annotation of the recovered MAGs showed that S-category genes (function unknown) were abundant in all genomes (Figure 7B). G-category genes (carbohydrate transport and metabolism) and J-category genes (translation, ribosomal structure, and biogenesis) were also highly represented across MAGs. *Unclassified Saccharopolyspora* contained the highest number of E-category genes (amino acid transport and metabolism), while *Saccharopolyspora rectivirgula* showed relatively high gene numbers across multiple functional categories (Figure S3). Only *Levilactobacillus brevis* lacked genes in the cell motility category (N).

### 3.8. Primary Metabolites and Secondary Metabolites of High-Quality MAG

The recovered high-quality MAGs showed diverse primary metabolic potentials, including fatty acid biosynthesis pathways and short-chain fatty acid (SCFA) fermentation modules (Figure 7C,D). In addition, molybdopterin-dependent oxidoreductases were widely detected in the recovered genomes. Gene clusters related to oxidative glycerol metabolism, pyruvate-to-acetate/formate conversion, and fumarate-to-succinate conversion were also identified in several MAGs. Among the recovered genomes, *Levilactobacillus brevis* contained a gallic acid degradation gene cluster and a putrescine-to-spermidine gene cluster. *Erwinia persicina* harbored a formate dehydrogenase gene cluster. Both *Erwinia persicina* and *Pantoea agglomerans* contained the SUC gene cluster and the PFL acetate gene cluster.

antiSMASH analysis revealed a diverse repertoire of secondary metabolite biosynthetic gene clusters (BGCs) across the high-quality MAGs. Among them, *Saccharopolyspora rectivirgula* showed the highest enrichment of secondary metabolic potential. NRPS-related clusters were the most abundant, particularly in MAG2, MAG20, and MAG14. In addition, hybrid clusters, multiple PKS-related clusters, and clusters associated with terpenes, carotenoids, and NI-siderophores were also detected.

## 4. Discussion

### 4.1. Microbial Community Analysis

Using metagenomic sequencing combined with metagenome-assembled genome analysis, this study profiled the previously uncharacterized microbial community and functional potential of mature HBQ, the starter of Highland barley Baijiu. The high Good’s coverage values indicated that the sequencing depth was sufficient to capture the microbial diversity of HBQ, while the similar alpha diversity observed among the parallel samples supported the reproducibility of the sampling strategy and suggested an overall stable community diversity pattern in mature HBQ. On this basis, the overall microbial structure of HBQ was further analyzed. The near-balanced proportion (1.22:1) between fungi and bacteria in HBQ is noteworthy. Similar patterns have been reported in certain high-temperature Daqu [20], in which the fungi-to-bacteria ratios were approximately 0.87:1 and 1.10:1 in white and yellow Daqu, respectively, whereas other Daqu types may show much more bacteria-dominated communities [21]. These comparisons suggest that the fungal/bacterial balance in Daqu is not fixed, but varies with Daqu type, fermentation temperature, and production conditions [22].

The HBQ microbiota was characterized by the marked predominance of *Lichtheimia ramosa* and *Pantoea agglomerans*, suggesting that these two species may provide an important structural and functional framework for the fungal and bacterial communities, respectively. This pattern is partly consistent with previous studies [23,24], in which *Lichtheimia* has been associated with saccharification-related functions in some Daqu types [25], while *Pantoea* has also been reported in low-temperature or light-flavor Baijiu-related starters and early fermentation environments [26]. At the same time, several fungi and bacteria were present at relatively low abundance; these non-dominant taxa should not be overlooked. Previous studies of Baijiu fermentation have shown that dominant microorganisms in Daqu do not necessarily remain dominant during grain fermentation, and some initially rare or low-abundance taxa may increase later under favorable acid, ethanol, or oxygen conditions [27,28]. Therefore, the HBQ community is more likely to represent a functionally layered microbial reservoir, in which dominant taxa support the core starter structure, while low-abundance taxa may serve as potential responders during subsequent fermentation and may help maintain ecological stability through microbial interactions and niche complementation.

The clustering pattern suggests that the microbial community of HBQ is more similar to that of light-flavor Baijiu Daqu than to those of other flavor-type Daqu included in this comparison [15]. However, HBQ still exhibited distinct taxonomic characteristics, particularly the relatively high abundances of *Lichtheimia* and *Pantoea*, together with several lactic acid bacteria and yeasts, indicating that HBQ represents a characteristic microbial assemblage rather than a simple variant of light-flavor Daqu. Compared with sauce-flavor Daqu, which showed relatively higher abundances of thermotolerant or spore-forming genera such as *Kroppenstedtia*, *Paenibacillus*, *Thermoactinomyces*, and *Virgibacillus* [13], HBQ was less enriched in these taxa and instead contained more *Lichtheimia*, *Pantoea*, *Leuconostoc*, and related genera. Strong-flavor Daqu showed comparatively higher abundances of *Acinetobacter*, *Rasamsonia*, and *Clavispora* [14], while feng-flavor Daqu was more strongly associated with *Saccharopolyspora*, *Bacillus*, *Streptomyces*, *Pichia*, and *Thermoascus* [16], suggesting that these starters possess distinct bacterial–fungal assemblages compared with HBQ. Chi-flavor Daqu was characterized by relatively high abundances of *Lactobacillus*, *Rhizopus*, *Thermomucor*, *Mucor*, and *Saccharomycopsis* [18], indicating a community structure with stronger enrichment of mucoralean fungi and lactobacilli than that observed in HBQ. In contrast, the abundance of *Saccharomyces* in HBQ was lower than that reported for rice-flavor Baijiu Daqu, in which *Saccharomyces* has been described as a dominant fungal genus [17]. Laobaigan-flavor Daqu showed comparatively higher abundances of *Isatchenkia* and *Enterobacter* [19], further indicating that each flavor-type starter harbors its own characteristic microbial signature. Several genera, including *Pichia*, *Rhizopus*, *Aspergillus*, *Weissella*, *Pediococcus*, *Staphylococcus*, and *Bacillus*, were shared across multiple flavor-type Daqu, suggesting that they may represent commonly occurring microbial groups in Baijiu starter ecosystems [29].

### 4.2. Functional Potential Analysis

The KEGG annotation results indicate that the functional potential of the HBQ microbiome is mainly associated with metabolic activities, especially carbohydrate metabolism and amino acid metabolism, indicating that these pathways serve as key drivers of the fermentation process. A similar functional profile has also been reported in the Chinese typical Daqu [30]. Carbohydrate metabolism is closely associated with starch degradation and glycolysis, thereby providing substrates and energy for alcoholic fermentation [31]. Amino acid metabolism contributes to the formation of higher alcohols and esters, which directly influence the aroma and taste of Baijiu in general [32]. The ko00290 pathway increased the concentrations of higher alcohols and ethyl esters in fermented Baijiu, which was crucial to endow it with floral and fruity aromas [33]. Meanwhile, about 20% of the functional contributions in the top 10 metabolic pathways could not be assigned to known taxa, suggesting that HBQ harbors numerous uncharacterized microbial resources. This may be related to the unique environment of the Qinghai–Tibet Plateau. The unannotated fraction also reflects limitations of current reference databases and indicates that the HBQ microbial ecosystem remains incompletely understood.

The eggNOG annotation indicated that the functional potential of the HBQ microbiome was concentrated mainly in carbohydrate metabolism and amino acid metabolism, which is consistent with the core requirements of the Baijiu fermentation process [34]. Additionally, the relatively high abundance of genes related to replication and repair suggests that microorganisms in HBQ may possess strong environmental adaptability, which could help them cope with stresses such as ethanol and acidity during fermentation [35]. In short, these functions may support alcohol production and flavor formation in Highland barley Baijiu. The GO annotation profile indicated that the HBQ microbiome was enriched in cellular and metabolic processes, catalytic activity, and binding, reflecting active substrate transformation during fermentation [36]. Transporter-related functions and categories associated with response to stimulus and biological regulation further suggest roles in nutrient transport and environmental adaptation [37,38]. Overall, these GO categories indicate functional potential related to substrate utilization, energy metabolism, and stress adaptation in HBQ.

In the CAZy database, the high abundance of GH suggests a strong capacity for polysaccharide degradation [39]. In particular, GH13 and GH15 may contribute to starch metabolism [40], whereas GH23/25 may be involved in microbial community regulation through lysozyme-related activity [25]. The high abundance of GT1 suggests glycosylation-related potential, while GH1 and GH3 may contribute to the release of bound aroma compounds through β-glucosidase activity [41]. In addition, cellulase- and pectinase-related families, including GH1, GH3, GH5, GH28, and GH43, suggest that HBQ can utilize structural polysaccharides in cereal raw materials and improve substrate accessibility [42]. Overall, the CAZy annotation indicates that HBQ has functional potential related to saccharification, microbial interaction, and flavor precursor transformation. MetaCyc annotation indicated that HBQ contained abundant pathways related to nucleoside and nucleotide biosynthesis, amino acid biosynthesis, and the synthesis of cofactors, carriers, and vitamins, suggesting broad metabolic capacity for microbial growth and adaptation during fermentation [43]. In addition, pathways associated with secondary metabolites, aromatic compounds, cell structures, and polyphenols were also detected, indicating potential roles in aroma formation and other bioactive functions in Baijiu [44].

### 4.3. Metabolic Network Predictions Analysis

The predicted metabolic network indicates that the microorganisms and enzymes in HBQ can metabolize the dominant flavor compounds in highland barley Baijiu. The flavor formation in Baijiu is closely associated with carbohydrate degradation and central carbon metabolism, with glucose, pyruvate, and acetyl-CoA serving as key metabolic nodes [45,46]. These intermediates connect substrate degradation with amino acid, fatty acid, and organic acid metabolism, thereby linking raw-material utilization to the biosynthesis of flavor-related compounds. In particular, the predicted involvement of the phenylalanine metabolism pathway suggests that aromatic amino acid metabolism may be an important route for the formation of floral and fruity compounds in this system. The incomplete enrichment of some downstream enzymes further suggests that flavor formation in Highland barley Baijiu may depend not only on the enzymatic potential of HBQ itself, but also on dynamic microbial succession during the multi-round brewing process.

Lipid and organic acid metabolism also appear to contribute to aroma development and flavor balance. Fatty acid elongation has been associated with the accumulation of lipid-derived volatile compounds in Baijiu [47], and the contribution of *Pantoea agglomerans* to lipid-related enzymes suggests that this species may participate in precursor formation. Previous studies have also reported a positive correlation between *Pantoea* abundance and ethyl acetate production during liquor fermentation [48]. In addition, the distribution of malate dehydrogenase, lactate dehydrogenase, and related enzymes among fungi, yeasts, and bacteria suggests that multiple microbial groups may cooperatively regulate organic acid transformation and ester precursor supply [49,50,51]. Overall, these results indicate that the flavor-related metabolic network of HBQ is jointly shaped by bacteria, yeasts, and filamentous fungi, with different taxa contributing to complementary branches of substrate conversion and aroma precursor formation.

Recovery of high-quality MAGs from complex fermented foods is difficult but informative for understanding functionally relevant microorganisms [52,53]. In this study, 42 medium-quality MAGs, including 9 high-quality genomes (6 after dereplication), were recovered from HBQs, and several unclassified groups were also detected, suggesting that the microbial diversity of HBQ remains incompletely characterized. CRISPR/Cas-related proteins were present in most MAGs, indicating potential genome defense capacity in many HBQ-associated microorganisms [54,55].

### 4.4. Metagenomic-Assembled Genomes Analysis

Metagenomic-assembled genomes may play important roles in Baijiu fermentation (Figure 7B). Recovery of a limited number of high-quality MAGs from HBQ is likely related to the high complexity, uneven abundance distribution, and strain-level diversity of this solid-state fermentation system. The abundance of S-category genes across MAGs indicates substantial uncharacterized functional potential in the HBQ metagenome, consistent with the complexity of fermentation microbiomes and incomplete database annotations. Enrichment of G-category genes highlights active carbohydrate transport and metabolism in HBQ microorganisms, supporting substrate degradation during fermentation [56]. Similarly, abundant J-category genes imply strong protein synthesis, sustaining microbial growth and metabolism. The high number of E-category genes in *Unclassified Saccharopolyspora* suggests its potential in amino acid transformation (Figure S3), which may contribute to ester and higher alcohol formation and flavor metabolism. Moreover, the broad functional profile of *Saccharopolyspora rectivirgula* indicates its multifunctional role in fermentation. The absence of cell motility genes (N) in *Levilactobacillus brevis* suggests reduced dependence on active motility, although its ecological role in repeated fermentation requires further study.

The recovered MAGs showed broad metabolic potential related to energy generation, substrate conversion, and redox balance, including pathways for fatty acid biosynthesis, SCFA fermentation, glycerol oxidation, pyruvate-to-acetate/formate conversion, and fumarate-to-succinate transformation. In particular, the presence of a gallic acid degradation gene cluster and a putrescine-to-spermidine gene cluster in *Levilactobacillus brevis* suggests potential roles in phenolic compound transformation and stress adaptation during fermentation [57,58]. In addition, the detection of formate dehydrogenase in *Erwinia persicina* and the SUC and PFL acetate gene clusters in both *E. persicina* and *Pantoea agglomerans* suggests that these bacteria may contribute to organic acid metabolism and acetate supply in HBQ, thereby potentially supporting ester precursor formation and subsequent aroma development. The antiSMASH results further indicated that the recovered MAGs possessed substantial secondary metabolic potential. The enrichment of NRPS, hybrid, and PKS-related clusters suggests that HBQ-associated microorganisms may produce structurally diverse bioactive compounds [59]. Carotenoids are important bacterial secondary metabolites, and their degradation products can contribute to aroma formation, including compounds such as β-damascon. In addition, terpene-, carotenoid-, and NI-siderophore-related clusters were also detected, indicating potential roles in aroma formation and microbial adaptation within the HBQ ecosystem [60,61,62]. Overall, genome-resolved analysis showed that high-quality MAGs recovered from HBQ encoded diverse primary and secondary metabolic functions potentially related to substrate utilization, stress adaptation, microbial interactions, and flavor precursor formation. The presence of unclassified taxa and unresolved functional genes suggests that HBQ still harbors incompletely characterized microbial resources, and further cultivation-based and functional validation will be needed to clarify their roles during Highland barley Baijiu fermentation.

However, because this study was based on a single batch of mature HBQ and a limited number of parallel subsamples, the generalizability of these findings remains constrained. Future studies should further investigate the dynamic behavior, microbial interactions, and functional expression of key populations during HBQ fermentation, in combination with multi-batch sampling and cultivation-based validation, to better understand the mechanisms underlying flavor formation and fermentation stability in highland barley Baijiu.

## 5. Conclusions

The microbial community composition and predicted functions of HBQ were revealed by Macrogenomics. The microbial community of HBQ shared some common features with other flavor-type Baijiu Daqu, while also exhibiting distinctive taxonomic characteristics that may reflect adaptation to the plateau environment. Based on functional annotations across multiple databases, HBQ exhibited strong metabolic potential related to Baijiu fermentation, particularly in pathways associated with carbohydrate metabolism, amino acid metabolism, and flavor precursor formation. There were abundant functional microorganisms and their enzyme profiles related to flavor substances. A total of 42 medium-quality MAGs, including 6 high-quality MAGs, were recovered from HBQ, providing genome-level insight into representative microbial populations in the HBQ ecosystem. The recovered MAGs encoded diverse primary and secondary metabolic functions potentially related to substrate utilization, stress adaptation, microbial interactions, and aroma formation. In addition, the presence of unclassified MAGs and unresolved functional fractions suggests that HBQ still harbors incompletely characterized microbial resources with potential ecological and functional importance. However, this study still has some limitations, such as the single batch and restricted sample size. Overall, this study provides a genomic basis for understanding the microbial functions of HBQ and offers theoretical support for improving HBQ quality and Highland barley Baijiu production.

## Data Availability

The original contributions presented in this study are included in the article and Supplementary Materials. Further inquiries can be directed to the corresponding authors. Raw metagenomic sequences were submitted to NCBI (National Center for Biotechnology Information) with project ID PRJNA1276891 (Contains 6 raw metagenomic sequencing files) and PRJNA1297374 (Contains 6 high-quality MAG files).

## Supplementary Materials

The following supporting information can be downloaded at: https://www.mdpi.com/article/10.3390/microorganisms14040877/s1. Figure S1: Sampling strategy for HBQ collection; Figure S2: Heatmaps of microbial community structure of HBQ and other previously analyzed Baijiu Daqu; Figure S3: Circular genome visualization of medium-quality MAGs (A), the functional annotation of MAGs microbiota genes by eggNOG (B), the gene number annotation of primary metabolites (C), secondary metabolites (E), and genomic mapping enabling visualization of gene distribution across contigs within each MAG, revealing regions that exhibit 100% similarity to known gene clusters (D: primary metabolites; F: secondary metabolites); Table S1: Statistics of sequencing and bioinformatics analysis; Table S2: Alpha diversity analysis of HBQ microbial community; Table S3: Details of classified medium-quality MAGs recovered from HBQ metagenomes; and Table S4: The details of CRISPR/Cas proteins detected from MAGs.

## Abbreviations

The following abbreviation is used in this manuscript:

HBQ	Highland barley Baijiu daQu
