# Supplementary material for: Metagenomics and Metagenome-Assembled Genomes Analysis of Highland Barley Baijiu Daqu
Source: Microorganisms. 2026 Apr 14;14(4):877. doi: 10.3390/microorganisms14040877 (PMC13118880; doi:10.3390/microorganisms14040877)
Supplement: Supplementary file 1 [file microorganisms-14-00877-s001.zip › microorganisms-4174955-supplementary.pdf]

Supplementary Material

Metagenomics and metagenome-assembled genomes analysis of highland barley Baijiu Daqu

Lihua Chen[a]*, Yuhang Chen[a], Qinghua Peng[a], Dingxia Zhou[a], Shengbao Feng[b]*

Author Affiliation(s):

a Faculty of Flavour Fragrance and Cosmetics, Shanghai Institute of Technology, Shanghai 201418, China

b Qinghai Huzhu Barley Wine Co., Ltd, Haidong, 810500, China

***Corresponding author:**

Lihua Chen, E-mail: lhchen@sit.edu.cn

Shengbao Feng, E-mail: Fengshengbao@qkj.com.cn

Co-author information:

Yuhang Chen, E-mail: 13739185299@163.com

Qinghua Peng, E-mail: pengqinghua0322@163.com

Dingxia Zhou, E-mail: 18930465101@163.com

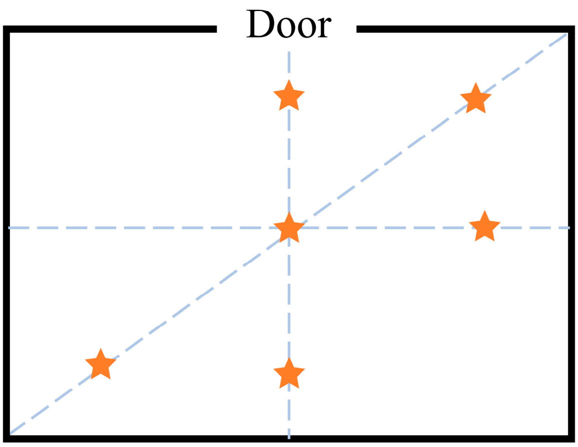

Figure S1. Sampling strategy for HBQ collection. Black box lines indicate the Daqu room, and five-pointed stars represent the sampling positions from which mature HBQ bricks were collected for metagenomic analysis.

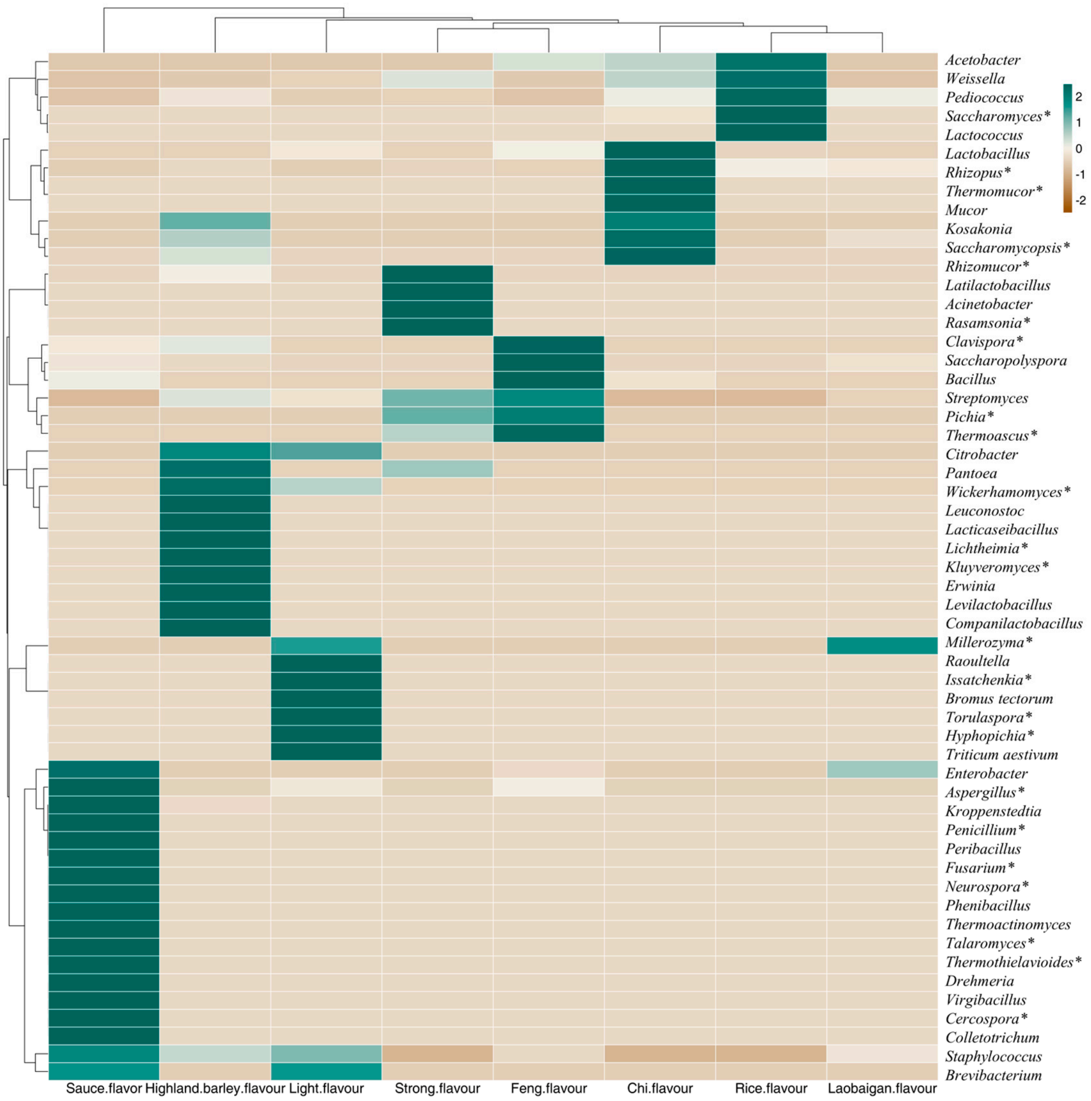

Figure S2. Heatmaps of microbial community structure of HBQ and other previously analysed Baijiu Daqu.

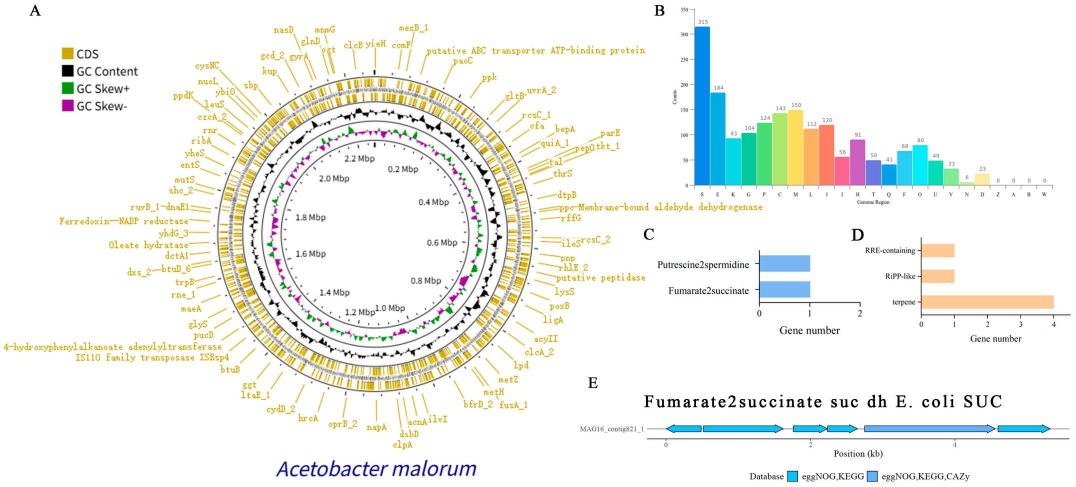

Acetobacter malorum

(Completeness=86.24%;Contamination=3.75%)

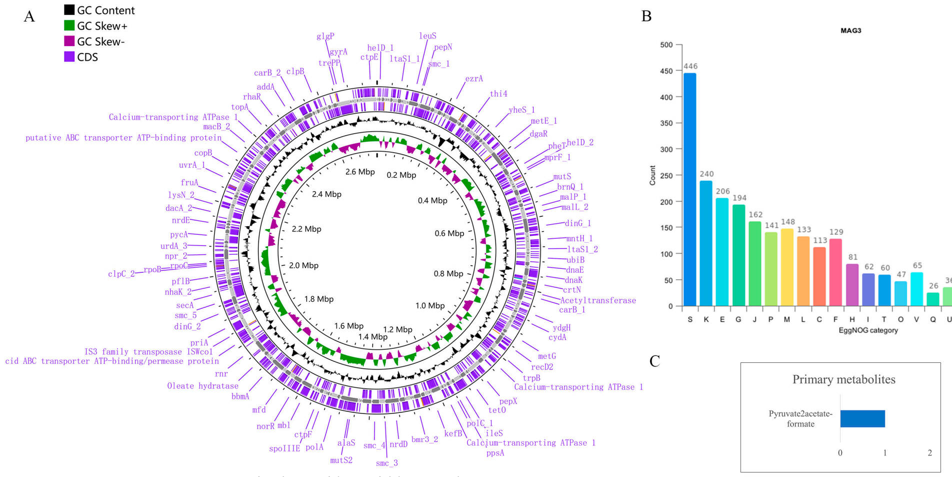

Lactiplantibacillus plantarum

(Completeness=96.19%; Contamination=7.73%)

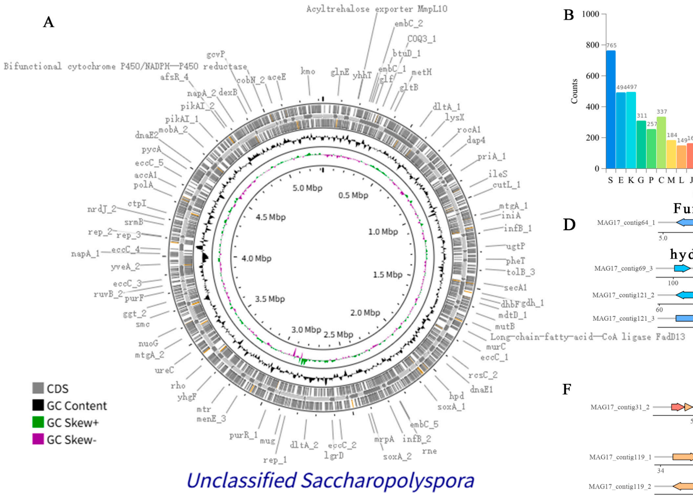

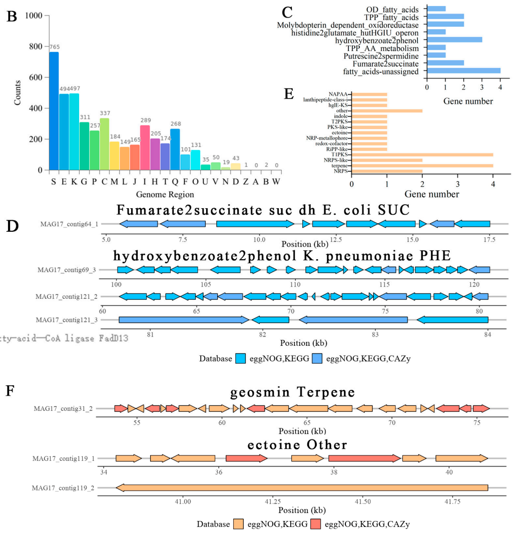

Unclassified Saccharopolyspora

(Completeness=85.70%;Contamination=3.56%)

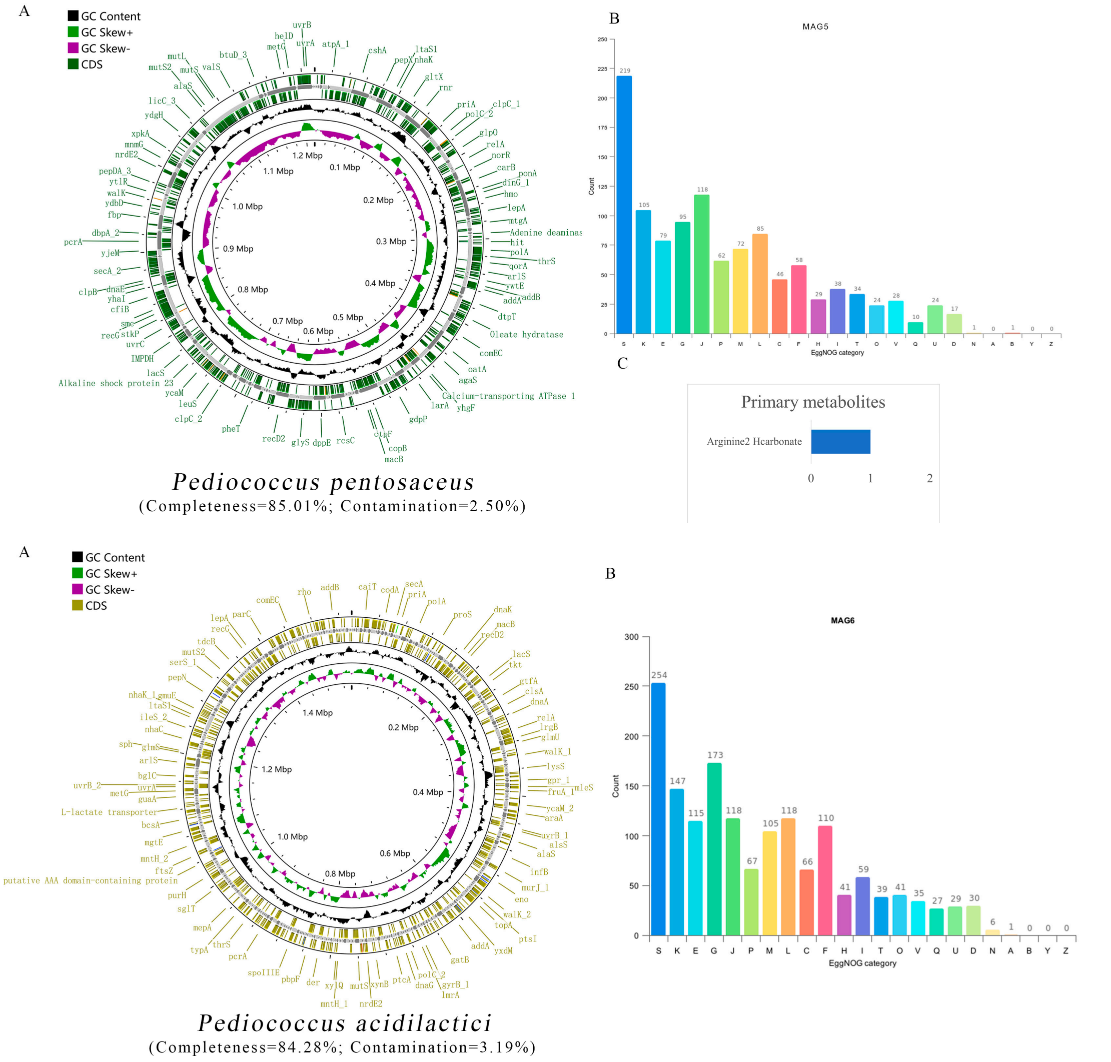

Figure S3. Circular genome visualization of medium-quality MAGs (A); The functional annotation of MAGs microbiota genes by eggNOG (B); The Gene number annotation of primary metabolites (C) and secondary metabolites (E); Genomic mapping enables visualization of gene distribution across contigs within each MAG, revealing regions that exhibit 100% similarity to known gene clusters (D: primary metabolites; F: secondary metabolites).

Table S1. Statistics of sequencing and bioinformatics analysis.

Parameter	Q1	Q2	Q3	Q4	Q5	Q6
**Sequencing**						
Raw data (Gb)	10.58	11.88	9.55	12.28	11.33	9.66
Clean data (Gb)	10.32	11.64	9.40	12.08	11.17	9.54
Clean Q20 (%)	99.12	98.86	99.07	99.05	99.10	99.15
Clean Q30 (%)	97.57	96.77	96.00	95.99	96.13	96.27
Clean GC (%)	37.01	37.19	44.06	43.53	42.81	41.93
Coverage (%)	97.51	97.97	98.49	98.37	98.58	98.69
**Assembly**						
Min seq length (bp)	300	300	300	300	300	300
Max seq length (bp)	253,333	305,043	189,778	228,126	260,288	233,313
Total seq number (bp)	229,051	178,012	197,148	230,050	233,844	205,867
Total seq length (kb)	314,425	297,073	269,692	286,799	279,021	255,442
N50 length (bp)	2,584	4,438	2729	1938	1813	2002
N90 length (bp)	490	523	494	483	476	482
**Gene Prediction**						
Number of ORFs	413,033	272,738	380,520	413,922	406,882	372,035
Annotated on KEGG	96,454	93,385	153,798	126,876	123,596	116,490
(% of ORFs)	(23.35%)	(34.24%)	(40.42%)	(30.65%)	(30.38%)	(31.31%)
Annotated on CAZy	48,840	47,429	70,431	57,198	55,576	52,433
(% of ORFs)	(11.82%)	(17.39%)	(18.51%)	(13.82%)	(13.66%)	(14.09%)
Annotated on Matecyc	27,378	26,681	41,551	34,975	34,291	32,870
(% of ORFs)	(6.63%)	(9.78%)	(10.92%)	(8.45%)	(8.43%)	(8.84%)
Annotated on eggNOG	169,507	163,737	270,139	222,169	216,606	202,634
(% of ORFs)	(41.04%)	(60.03%)	(70.99%)	(53.67%)	(53.24%)	(54.47%)
Annotated on GO	3,980	3,841	7,738	6,079	6,119	5,661
(% of ORFs)	(0.96%)	(1.41%)	(2.03%)	(1.47%)	(1.50%)	(1.52%)

Note：Functional annotations from different databases were individually counted; ORFs denote the predicted genes.

Table S2. Alpha diversity analysis of HBQ microbial community.

Alpha diversity index	Mean ± Standard Deviation
Chao1	2743.2 ± 359.8
Goods coverage	0.999718 ± 0.000199
Simpson	0.9189 ± 0.0270
Pielou e	0.4719 ± 0.0331
Shannon	3.683 ± 0.274
Observed species	2495.0 ± 502.6
ACE	2709.7 ± 379.0

Table S3. Details of classified medium-quality MAGs recovered from HBQ metagenomes.

MAGs-id	lineage	Contigs Num	Genome size (kb)	Longest contig (kb)	N50 contigs (kb)	Mean contig length (kb)	GC	Completeness (%)	Contamination (%)	Binner
**MAG1**	***Unclassified Kroppenstedtia***	565	3226.794	48.482	10.633	5.711	0.515	98.78	4.102	Q3
**MAG2**	***Erwinia persicina***	644	5344.669	115.526	23.311	8.299	0.558	98.38	1.760	Q3
**MAG4**	***Levilactobacillus brevis***	215	2004.931	72.62	20.768	9.325	0.464	91.97	4.375	Q3
MAG6	*Bacillus licheniformis*	1080	3180.85	14.63	3.595	2.945	0.473	79.38	1.798	Q3
MAG9	*Erwinia gerundensis*	1793	3372.819	8.707	1.962	1.881	0.567	64.90	9.775	Q3
MAG10	*Pseudomonas fluorescens*	1837	3280.243	9.026	1.825	1.785	0.617	53.26	2.791	Q3
**MAG12**	***Unclassified Kroppenstedtia***	710	3084.351	37.222	6.363	4.344	0.517	100.00	4.538	Q4
**MAG14**	***Pantoea agglomerans***	881	4117.984	29.346	6.99	4.674	0.564	95.77	4.499	Q4
MAG16	*Acetobacter malorum*	894	2266.456	12.899	2.926	2.535	0.595	86.24	3.747	Q4
MAG17	*Unclassified Saccharopolyspora*	133	5324.221	201.429	56.591	40.031	0.722	85.70	3.557	Q4
**MAG20**	***Saccharopolyspora rectivirgula***	76	4039.311	260.288	107.633	53.148	0.690	99.17	1.090	Q5
**MAG22**	***Unclassified Kroppenstedtia***	968	2739.755	21.979	3.511	2.83	0.520	95.29	1.923	Q5
MAG29	*Unclassified Brevibacterium*	1251	2472.914	8.989	2.086	1.976	0.651	62.15	0.000	Q5
**MAG33**	***Leuconostoc citreum***	127	1728.023	123.297	34.299	13.606	0.392	98.92	1.516	Q1
**MAG34**	***Levilactobacillus brevis***	231	2302.175	122.98	32.567	9.966	0.465	98.43	1.458	Q1
MAG35	*Lactiplantibacillus plantarum*	325	2759.012	50.028	16.615	8.489	0.454	96.19	7.733	Q1
MAG37	*Pediococcus pentosaceus*	156	1215.279	78.495	18.378	7.79	0.370	85.01	2.503	Q1
MAG38	*Pediococcus acidilactici*	555	1538.71	23.463	3.579	2.772	0.428	84.28	3.189	Q2
MAG39	*Leuconostoc mesenteroides*	234	1053.789	21.874	7.043	4.503	0.384	66.65	2.116	Q2
MAG40	*Staphylococcus succinus*	1575	2928.177	13.938	1.925	1.859	0.325	65.23	8.665	Q2
MAG41	*Companilactobacillus crustorum*	37	739.856	73.91	34.975	19.996	0.349	57.05	0.000	Q2

Note: The MAGs retained after 99% redundancy removal by the dRep software, and high-quality MAGs-id have been bolded.

Table S4. The details of CRISPR/Cas proteins detected from MAGs

MAG_id	Contig_id	Crispr_id	Start	End	Number of spacers	Length(bp)
MAG1	contig28	Crispr1	307	378	1	72
MAG1	contig79	Crispr2	57	994	10	938
MAG1	contig99	Crispr3	1488	1608	2	121
MAG1	contig145	Crispr4	11136	11350	3	215
MAG1	contig145	Crispr5	11595	12358	11	764
MAG1	contig149	Crispr6	636	728	1	93
MAG1	contig162	Crispr7	744	1534	8	791
MAG1	contig178	Crispr8	7545	7842	5	298
MAG1	contig209	Crispr9	6496	6616	2	121
MAG1	contig210	Crispr10	4657	4780	2	124
MAG1	contig230	Crispr11	297	370	1	74
MAG1	contig237	Crispr12	4427	4525	1	99
MAG1	contig246	Crispr13	5736	5905	3	170
MAG1	contig266	Crispr14	875	1132	3	258
MAG1	contig328	Crispr15	289	650	4	362
MAG1	contig342	Crispr16	105	604	7	500
MAG1	contig365	Crispr17	10081	10204	2	124
MAG1	contig411	Crispr18	4649	4726	1	78
MAG1	contig411	Crispr19	12247	12322	1	76
MAG1	contig423	Crispr20	1	74	1	74
MAG1	contig450	Crispr21	109	235	2	127
MAG1	contig482	Crispr22	3642	3731	1	90
MAG1	contig488	Crispr23	9	82	1	74
MAG1	contig488	Crispr24	129	251	2	123
MAG1	contig498	Crispr25	1175	1760	7	586
MAG2	contig10	Crispr1	7334	7431	1	98
MAG2	contig336	Crispr2	11471	11875	5	405
MAG2	contig355	Crispr3	26185	26426	4	242
MAG2	contig605	Crispr4	27647	27816	2	170
MAG3	contig17	Crispr1	5931	6032	1	102
MAG3	contig142	Crispr2	804	1119	5	316
MAG3	contig254	Crispr3	3279	3474	2	196
MAG3	contig455	Crispr4	3379	3589	2	211
MAG3	contig480	Crispr5	2238	2330	1	93
MAG3	contig738	Crispr6	6224	6326	1	103
MAG4	contig94	Crispr1	4790	4936	2	147
MAG4	contig180	Crispr2	50717	51110	6	394
MAG4	contig209	Crispr3	43	192	2	150
MAG6	contig496	Crispr1	135	1213	15	1079
MAG6	contig496	Crispr2	1294	1375	1	82

MAG_id	Contig_id	Crispr_id	Start	End	Number of spacers	Length(bp)
MAG6	contig539	Crispr3	2420	2586	2	167
MAG6	contig816	Crispr4	1709	2372	7	664
MAG6	contig946	Crispr5	5121	5224	1	104
MAG7	contig79	Crispr1	97	310	3	214
MAG8	contig182	Crispr1	1670	2056	4	387
MAG8	contig329	Crispr2	617	1057	5	441
MAG9	contig10	Crispr1	32	226	2	195
MAG9	contig16	Crispr2	141	234	1	94
MAG9	contig95	Crispr3	5833	5920	1	88
MAG9	contig673	Crispr4	601	790	3	190
MAG9	contig692	Crispr5	196	269	1	74
MAG9	contig838	Crispr6	62	183	1	122
MAG9	contig1090	Crispr7	1	74	1	74
MAG9	contig1635	Crispr8	764	1013	3	250
MAG9	contig1646	Crispr9	1395	1510	1	116
MAG9	contig1693	Crispr10	287	381	1	95
MAG10	contig87	Crispr1	1385	1481	1	97
MAG11	contig266	Crispr1	3001	3100	1	100
MAG12	contig22	Crispr1	1	72	1	72
MAG12	contig32	Crispr2	47	270	4	224
MAG12	contig38	Crispr3	4800	5204	7	405
MAG12	contig66	Crispr4	3299	3933	9	635
MAG12	contig86	Crispr5	198	275	1	78
MAG12	contig87	Crispr6	5696	6047	4	352
MAG12	contig163	Crispr7	5381	5455	1	75
MAG12	contig190	Crispr8	211	986	10	776
MAG12	contig224	Crispr9	526	597	1	72
MAG12	contig239	Crispr10	11721	11796	1	76
MAG12	contig285	Crispr11	13681	13752	1	72
MAG12	contig331	Crispr12	1945	2068	2	124
MAG12	contig334	Crispr13	6	949	13	944
MAG12	contig389	Crispr14	1	74	1	74
MAG12	contig410	Crispr15	190	287	1	98
MAG12	contig494	Crispr16	9	129	2	121
MAG12	contig522	Crispr17	88	650	8	563
MAG12	contig523	Crispr18	871	1099	3	229
MAG12	contig662	Crispr19	392	512	2	121
MAG13	contig5	Crispr1	46085	46478	6	394
MAG13	contig73	Crispr2	482	578	1	97
MAG13	contig84	Crispr3	2340	2733	6	394
MAG13	contig104	Crispr4	2114	2216	1	103

MAG_id	Contig_id	Crispr_id	Start	End	Number of spacers	Length(bp)
MAG13	contig218	Crispr5	776	921	2	146
MAG13	contig301	Crispr6	3198	3513	5	316
MAG14	contig45	Crispr1	2683	2945	3	263
MAG14	contig71	Crispr2	4020	4301	3	282
MAG14	contig118	Crispr3	2108	2420	5	313
MAG14	contig290	Crispr4	5953	6052	1	100
MAG14	contig298	Crispr5	6948	7158	2	211
MAG15	contig249	Crispr1	345	558	3	214
MAG16	contig230	Crispr1	737	957	3	221
MAG16	contig487	Crispr2	2270	2754	5	485
MAG16	contig742	Crispr3	1	207	2	207
MAG17	contig1	Crispr1	15802	15901	1	100
MAG17	contig4	Crispr2	4470	4556	1	87
MAG17	contig6	Crispr3	94506	94588	1	83
MAG17	contig39	Crispr4	141	273	1	133
MAG17	contig39	Crispr5	42850	42979	2	130
MAG17	contig42	Crispr6	21356	21433	1	78
MAG17	contig50	Crispr7	31153	31275	1	123
MAG17	contig56	Crispr8	48849	48928	1	80
MAG17	contig64	Crispr9	8337	8462	2	126
MAG17	contig69	Crispr10	17	98	1	82
MAG17	contig72	Crispr11	7497	7627	2	131
MAG17	contig76	Crispr12	25913	25990	1	78
MAG17	contig78	Crispr13	17170	17245	1	76
MAG17	contig81	Crispr14	44798	44975	3	178
MAG17	contig82	Crispr15	1182	1256	1	75
MAG17	contig84	Crispr16	27504	27683	2	180
MAG17	contig86	Crispr17	15081	15156	1	76
MAG17	contig89	Crispr18	69281	69462	3	182
MAG17	contig104	Crispr19	1	190	3	190
MAG17	contig121	Crispr20	63198	63279	1	82
MAG18	contig6	Crispr1	6033	6114	1	82
MAG18	contig7	Crispr2	1546	1627	1	82
MAG18	contig7	Crispr3	2735	2826	1	92
MAG18	contig18	Crispr4	14034	14131	1	98
MAG18	contig22	Crispr5	10972	11076	1	105
MAG18	contig22	Crispr6	34678	34769	1	92
MAG18	contig39	Crispr7	3105	3196	1	92
MAG18	contig39	Crispr8	29005	29076	1	72
MAG18	contig39	Crispr9	40492	40599	1	108
MAG18	contig55	Crispr10	15901	15989	1	89

MAG_id	Contig_id	Crispr_id	Start	End	Number of spacers	Length(bp)
MAG18	contig55	Crispr11	38303	38388	1	86
MAG18	contig59	Crispr12	2008	2093	1	86
MAG18	contig66	Crispr13	2107	2191	1	85
MAG18	contig66	Crispr14	15704	15792	1	89
MAG18	contig71	Crispr15	66361	66438	1	78
MAG18	contig72	Crispr16	15039	15136	1	98
MAG18	contig77	Crispr17	15345	15436	1	92
MAG18	contig93	Crispr18	3522	3684	2	163
MAG18	contig93	Crispr19	3735	3977	4	243
MAG18	contig94	Crispr20	161	353	2	193
MAG18	contig120	Crispr21	29449	29520	1	72
MAG18	contig124	Crispr22	24884	25064	2	181
MAG18	contig124	Crispr23	29974	30067	1	94
MAG18	contig124	Crispr24	41229	41632	5	404
MAG18	contig134	Crispr25	15788	15905	1	118
MAG18	contig134	Crispr26	25921	25994	1	74
MAG18	contig140	Crispr27	3496	3618	1	123
MAG18	contig156	Crispr28	56321	56402	1	82
MAG18	contig164	Crispr29	6162	6243	1	82
MAG19	contig111	Crispr1	4425	4528	1	104
MAG19	contig504	Crispr2	857	975	1	119
MAG19	contig526	Crispr3	93	361	4	269
MAG19	contig705	Crispr4	2450	2710	3	261
MAG20	contig2	Crispr1	27529	28007	6	479
MAG20	contig30	Crispr2	12609	12843	3	235
MAG20	contig46	Crispr3	2278	2609	5	332
MAG20	contig46	Crispr4	5829	6232	6	404
MAG20	contig58	Crispr5	4	336	5	333
MAG20	contig71	Crispr6	101298	101423	1	126
MAG20	contig74	Crispr7	99002	99122	1	121
MAG21	contig67	Crispr1	21601	21687	1	87
MAG21	contig90	Crispr2	6065	6164	1	100
MAG21	contig123	Crispr3	812	1009	2	198
MAG21	contig335	Crispr4	2104	2418	5	315
MAG21	contig546	Crispr5	526	736	2	211
MAG21	contig565	Crispr6	1881	1988	1	108
MAG21	contig583	Crispr7	83	272	2	190
MAG22	contig68	Crispr1	1467	1571	1	105
MAG22	contig142	Crispr2	2714	2868	2	155
MAG22	contig170	Crispr3	2936	3230	5	295
MAG22	contig218	Crispr4	3142	3423	4	282

MAG_id	Contig_id	Crispr_id	Start	End	Number of spacers	Length(bp)
MAG22	contig231	Crispr5	41	116	1	76
MAG22	contig384	Crispr6	2296	2460	2	165
MAG22	contig496	Crispr7	9	129	2	121
MAG22	contig499	Crispr8	49	172	2	124
MAG22	contig601	Crispr9	233	975	8	743
MAG22	contig618	Crispr10	3383	4046	8	664
MAG22	contig625	Crispr11	361	483	2	123
MAG22	contig764	Crispr12	1049	1120	1	72
MAG22	contig767	Crispr13	1	121	2	121
MAG22	contig854	Crispr14	2097	2168	1	72
MAG22	contig857	Crispr15	385	458	1	74
MAG23	contig96	Crispr1	2135	2378	4	244
MAG23	contig118	Crispr2	995	1195	2	201
MAG23	contig186	Crispr3	19583	19680	1	98
MAG23	contig190	Crispr4	224	1290	17	1067
MAG23	contig199	Crispr5	52	335	3	284
MAG23	contig334	Crispr6	55	332	3	278
MAG24	contig75	Crispr1	601	1074	6	474
MAG24	contig725	Crispr2	188	564	5	377
MAG26	contig67	Crispr1	4375	4690	5	316
MAG26	contig132	Crispr2	226	327	1	102
MAG26	contig478	Crispr3	161	260	1	100
MAG26	contig687	Crispr4	3221	3416	2	196
MAG26	contig800	Crispr5	2338	2548	2	211
MAG27	contig16	Crispr1	72	404	5	333
MAG27	contig138	Crispr2	128	409	3	282
MAG27	contig142	Crispr3	625	1028	5	404
MAG28	contig254	Crispr1	885	1229	4	345
MAG28	contig264	Crispr2	687	972	4	286
MAG28	contig371	Crispr3	553	652	1	100
MAG30	contig383	Crispr1	235	478	4	244
MAG30	contig874	Crispr2	2121	2213	1	93
MAG32	contig146	Crispr1	3289	3424	2	136
MAG32	contig206	Crispr2	2297	2370	1	74
MAG32	contig229	Crispr3	7038	7123	1	86
MAG32	contig391	Crispr4	3356	3463	1	108
MAG32	contig469	Crispr5	933	1084	2	152
MAG32	contig486	Crispr6	1008	1089	1	82
MAG32	contig493	Crispr7	1080	1292	2	213
MAG32	contig704	Crispr8	1606	1848	4	243
MAG32	contig757	Crispr9	609	680	1	72

MAG_id	Contig_id	Crispr_id	Start	End	Number of spacers	Length(bp)
MAG32	contig820	Crispr10	1332	1423	1	92
MAG32	contig858	Crispr11	893	986	1	94
MAG32	contig962	Crispr12	1995	2078	1	84
MAG32	contig968	Crispr13	37	108	1	72
MAG32	contig1049	Crispr14	7625	7696	1	72
MAG32	contig1072	Crispr15	2468	2543	1	76
MAG32	contig1096	Crispr16	3953	4044	1	92
MAG33	contig_7	Crispr1	760	872	1	113
MAG33	contig_8	Crispr2	1993	2169	3	177
MAG33	contig_32	Crispr3	18600	19237	9	638
MAG33	contig_91	Crispr4	867	984	1	118
MAG33	contig_100	Crispr5	914	994	1	81
MAG33	contig_100	Crispr6	1144	1332	2	189
MAG34	contig_1	Crispr1	21135	21467	5	333
MAG34	contig_157	Crispr2	231	624	6	394
MAG35	contig_20	Crispr1	36	633	7	598
MAG35	contig_81	Crispr2	29735	29946	3	212
MAG35	contig_162	Crispr3	4931	5241	4	311
MAG35	contig_171	Crispr4	1974	2302	4	329
MAG35	contig_271	Crispr5	33040	33355	5	316
MAG35	contig_288	Crispr6	12561	12822	3	262
MAG36	contig_312	Crispr1	2906	3221	5	316
MAG36	contig_444	Crispr2	165	272	1	108
MAG36	contig_746	Crispr3	281	380	1	100
MAG38	contig_126	Crispr1	1003	1092	1	90
MAG38	contig_296	Crispr2	1	118	1	118
MAG38	contig_467	Crispr3	2635	2780	2	146
